# Complete Terahertz Polarization Control with Broadened Bandwidth via Dielectric Metasurfaces

**DOI:** 10.1186/s11671-021-03614-y

**Published:** 2021-10-19

**Authors:** Dacheng Wang, Song Sun, Zheng Feng, Wei Tan

**Affiliations:** 1 Microsystem and Terahertz Research Center, CAEP, Chengdu, 610200 China; 2 Institute of Electronic Engineering, CAEP, Mianyang, 621999 China

**Keywords:** Terahertz, Dielectric metasurfaces, Polarization control, Ellipticity

## Abstract

We demonstrate terahertz dielectric metasurfaces with anisotropic multipoles within the framework of the generalized Huygens principle, in which the interference among these multipoles achieves giant phase shift with broadened bandwidth and high transmission coefficients. More importantly, owing to the anisotropic design, various phase delays between *π*/2 and 3*π*/2 are obtained, which convert the incident linearly polarized terahertz wave into right/left-handed circularly polarized light, elliptically polarized light and cross-polarized light. Both simulation and experimental results verify complete terahertz polarization control with the ellipticity ranging from 1 to − 1, which paves a way for polarization-related applications of terahertz meta-devices.

**Supplementary Information:**

The online version contains supplementary material available at 10.1186/s11671-021-03614-y.

## Introduction

Polarization represents one of the key parameters that quantify the state of electromagnetic wave [1]. Particularly, polarization control in terahertz region has attracted great research interest due to potential applications in terahertz technology [2, 3]. However, terahertz wave generated from most terahertz sources is linearly polarized [4], which cannot fulfill the requirement in complex polarimetric terahertz systems. The conventional approaches to manipulate the polarization of terahertz wave involve birefringent materials, which inherently suffer from many disadvantages, including bulky size and narrow band operation. Such drawbacks hinder these devices being integrated into modern compact and broadband terahertz photonic systems.

In recent years, metasurfaces, which are artificially engineered antenna arrays, enable an efficient approach to mold the polarization of terahertz wave [5, 6]. Various metallic metasurfaces have been proposed to realize polarization control. The proposed designs are normally based on anisotropic metasurfaces [7], chiral metasurfaces [8] and multilayer metasurfaces [9, 10]. Active media, such as phase change materials [11], semiconductors [8], two dimensional materials [12, 13], liquid crystals [14] and superconductors [15], have been integrated into metasurfaces to extend the functionalities. Multilayer and active metallic metasurfaces can further enhance the performance of polarization control with the sacrifice of high losses and complex fabrication process. Recently, dielectric metasurfaces, composed by dielectric antennas, provide a new approach to control electromagnetic wave [16]. Assisted by the interference between electric and magnetic Mie resonances, dielectric metasurfaces are capable of realizing 2*π* phase control with high efficiency [17]. Great efforts have been committed to enhancing the performance of dielectric metasurfaces for terahertz polarization control [18, 19]. However, most previous reported works are based on electric and magnetic dipole resonances, which realized limited performance, such as limited phase delay control ranges and in principle single-frequency operation [17], and thus hindered the complete manipulation of the polarization of terahertz wave with high performance.

Here, we propose dielectric metasurfaces with multipoles, which greatly lift the phase dispersion with the phase shift up to 4*π* and realize giant phase delay, broadened bandwidth and high efficiency, enabling complete terahertz polarization control. Composed by elliptical silicon pillar arrays, the proposed metasurfaces are capable of supporting different electric and magnetic multipoles. By overlapping these multipoles, near perfect transmission in broadened bandwidth and up to 4*π* phase shift can be achieved utilizing generalized Huygens principle [20, 21]. Owing to the anisotropy of the silicon pillars, the superposition of multipoles can be independently altered along the short and long axes of the elliptical pillars. Thus, giant phase delay in a broadband is achievable in such dielectric metasurfaces, which shows superior performance compared to other metallic and dielectric designs (see Additional file 1: Fig. S1). Since our proposed designs can achieve complete polarization control within a simple design framework, the meta-atoms can be artificially arranged to spatially vary the degree of polarization and generate complex terahertz beam, such as ellipticity-variant vector fields [22].

## Design and Simulation

Electromagnetic wave scattered from a dielectric antenna can be decomposed into multipoles with different symmetries [23]. When the dielectric antenna is arranged into arrays in metasurfaces, the scattered field $\overline{E}$ can be expressed as a sum of a symmetric component $\overline{E}_{s}$ and an anti-symmetric component $\overline{E}_{as}$. Thus, the transmission and reflection of the metasurfaces along the wave propagating direction $\hat{z}$ can be generally derived as [21, 24, 25]:

1 $$T = \left| {1 + \overline{E}_{s} (\hat{z}) + \overline{E}_{as} (\hat{z})} \right|^{2} ,$$

2 $$R = \left| {\overline{E}_{s} ( - \hat{z}) + \overline{E}_{as} ( - \hat{z})} \right|^{2} ,$$

where the amplitude of the incident wave is defined as 1. In order to realize high transmission and negligible reflection, $\overline{E}_{s} ( - \hat{z})$ and $\overline{E}_{as} ( - \hat{z})$ in the backward direction should have the same amplitudes but opposite phases. Particularly, when the dielectric antenna supports two multipoles, such as a symmetric resonance (e.g. electric dipole) and an anti-symmetric resonance (e.g. magnetic dipole), their superposition can satisfy the requirement of destructive interference. The destructive interference leads to zero reflection when these two dipole modes possess the same resonance frequency with the same amplitude and quality factor, which has been proposed in Huygens metasurfaces [17]. However, such destructive interference only occurs in a narrow band, which fundamentally imposes restrictions on the realization of broadband devices. To broaden the operating bandwidth, the scattered fields $\overline{E}_{s}$ and $\overline{E}_{as}$ should include the contributions from other high order multipoles, where the resultant transmission is a balance of multipolar interference among these multipoles. This scenario resembles to the concept of generalized Kerker condition with multipolar interference [26–28].

**Fig. 1:**
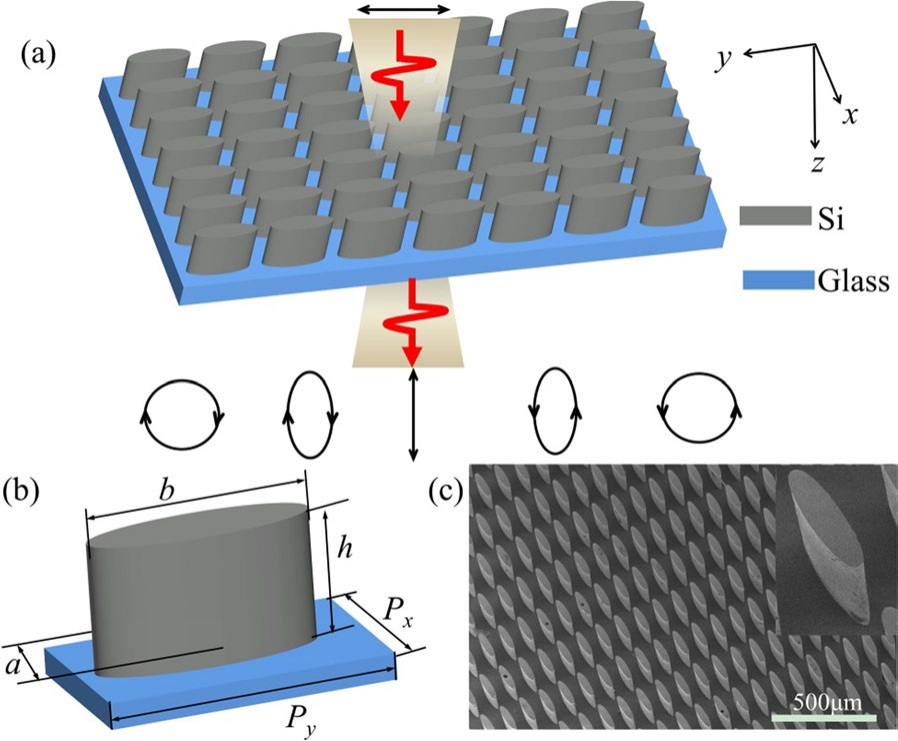
**a** Schematic of the dielectric metasurfaces, which realize full polarization control. **b** Unit cell of the dielectric metasurfaces. **c** SEM image of typical silicon pillar arrays in a tilted view with a zoom-in image

To fully cover all the polarization states, including right/left-handed circular polarization, elliptical polarization and linear polarization, the phase delay should cover from 90° to 270°, which corresponds to the ellipticity ranging from 1 to − 1. We thereby propose anisotropic dielectric metasurfaces composed by elliptical silicon pillar arrays, as shown in Fig. 1a. In the terahertz region, intrinsic silicon is adopted to eliminate absorption losses. As indicated in Fig. 1a, the linearly polarized incident light can be converted into circularly polarized light, elliptically polarized light and cross-polarized light, when multipolar interference maintains different combinations with respect to different geometry sizes. Figure 1b shows the parameters of the unit cell. The lengths of the short and long axes of the elliptical pillar are *a* and *b*, respectively. The height of the pillar is *h*. The periodicities of the unit cell along the short and long axes are *P*_*x*_ and *P*_*y*_, respectively. Figure 1c shows the scanning electron microscope (SEM) image of typical silicon pillar arrays, which will be discussed in the methods section.

**Fig. 2:**
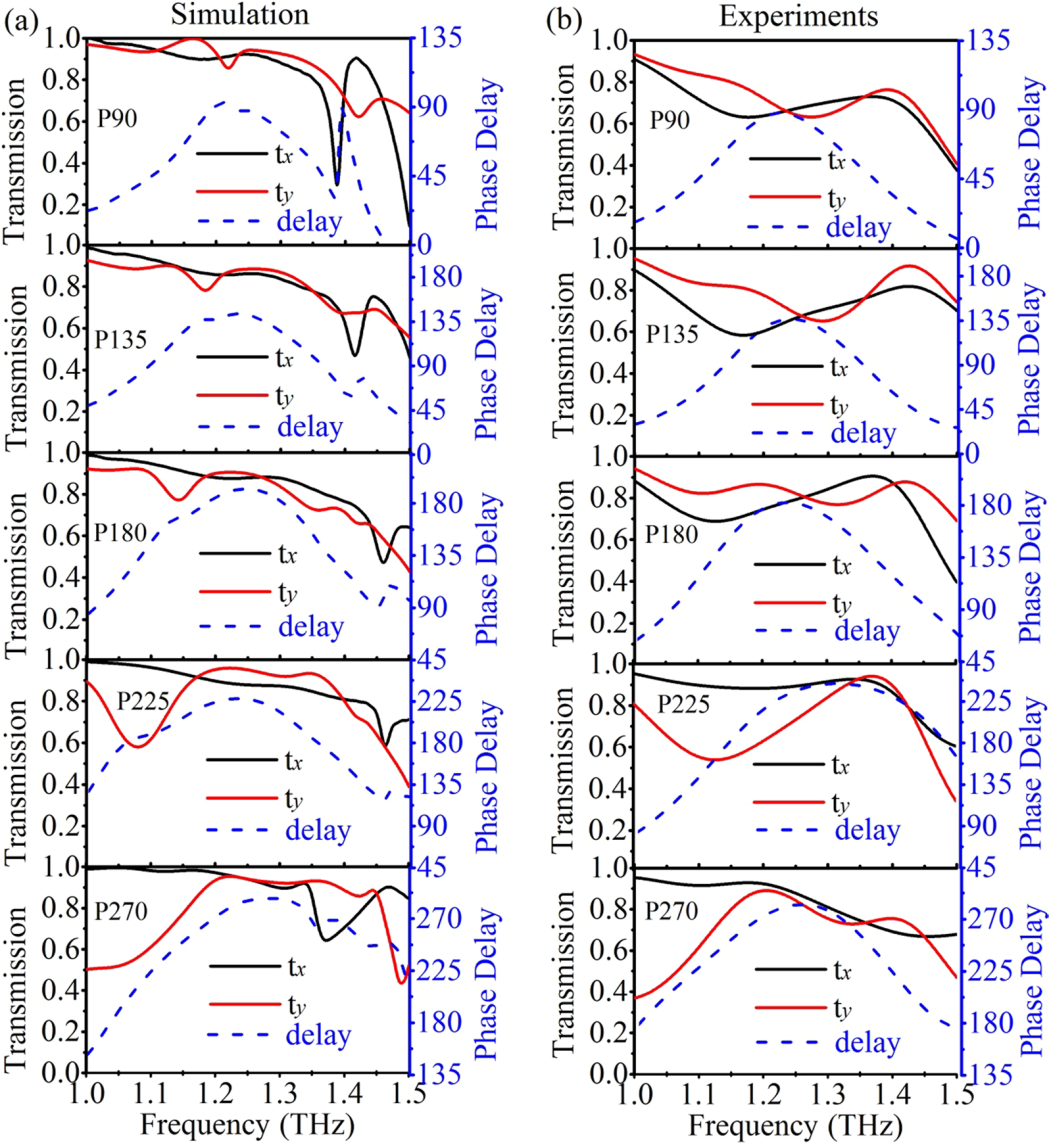
**a** Simulated and **b** experimental measured transmission coefficients and phase delays of the dielectric metasurfaces for the designs of P90, P135, P180, P225 and P270, respectively

To realize complete terahertz polarization control in the proposed dielectric metasurfaces, numerical simulation is carried out to optimize the dimensions of the dielectric metasurfaces, which simultaneously meet the requirement of 90° to 270° phase delay variation with high transmission and broadened bandwidth. Between 90° and 270°, a step size of 45° is chosen to demonstrate different polarization control schemes. Here, we name different designs regarding to their phase delays, which are defined as P90, P135, P180, P225 and P270, respectively. We performed the numerical simulation in the commercial simulation software CST microwave studio. In the simulation, silicon is treated as a lossless dielectric with *ε*_Si_ = 11.7 in the terahertz region. The substrate is modeled as a lossless dielectric with ε_sub_ = 4.5. Periodic boundary conditions are applied along both *x*- and *y*-axes. Terahertz wave is irradiated on the pillars in the z direction with open add space boundary condition. On the back side of the substrate, an open boundary condition is adopted to simulate a semi-infinite substrate. Figure 2a shows the simulated transmission and phase delays of five different metasurfaces. The detailed parameters of all the metasurfaces are presented in Additional file 1: Table S1. It is observed that all the metasurfaces manifest high transmission coefficients for both *x*- and *y*-polarized incidences from 1.2 to 1.3 THz, while the phase delays vary from 90°, 135°, 180°, 225° to 270°, respectively. The equal transmission coefficients with phase delay of 90° indicate that the incident light can be converted into a left-handed circularly polarized (LCP) light. Similarly, the phase delays of 135°, 180°, 225° and 270° are obtained with the polarization of the output light covering elliptical, cross and right-handed circular polarization. Thus, complete polarization control of terahertz wave is accomplished in the proposed dielectric metasurfaces, which shows superior performance compared to those meta-devices with limited bandwidths, low efficiencies and limited coverage of phase delays [18, 29].

## Results and Discussion

To validate the performance of polarization control, the silicon pillar arrays were fabricated and characterized in a terahertz time domain spectroscope (THz-TDS). The fabrication process can be found in the Methods section. A thin borosilicate glass (BF33, thickness 300 μm) is chosen as the substrate. The SEM image of a typical sample for the design with 270°phase delay is presented in Fig. 1c in a tilted view with a zoomed-in image as the inset. To characterize the performance of the metasurfaces, the electric fields of the transmitted terahertz wave along the short and long axes of the silicon pillars were denoted as $\overline{E}_{x}$ and $\overline{E}_{y}$. A bare glass substrate was measured as a reference with the corresponding transmitted electric fields of $\overline{E}_{x({\rm ref})}$ and $\overline{E}_{y({\rm ref})}$. The transmission coefficients were expressed as $\overline{t}_{x} = \overline{E}_{x} /\overline{E}_{x({\rm ref})}$ and $\overline{t}_{y} = \overline{E}_{y} /\overline{E}_{y({\rm ref})}$. The phase delay between two orthogonal polarizations was calculated and denoted as $\varphi = \varphi_{y} - \varphi_{x} = \arg (\overline{t}_{y} ) - \arg (\overline{t}_{x} )$. The details of the measurement system can be found in the Methods section.

The measured transmission coefficients and phase delays of the dielectric metasurfaces are shown in Fig. 2b. As can be seen, high transmission coefficients within the designed frequency ranges are obtained for the cases of P90, P135, P180, P225 and P270, with the corresponding phase delays close to 90°, 135°, 180°, 225° and 270°, respectively. A small discrepancy between the simulated and measured results can be observed, which may originate from the size fluctuation during the fabrication process. Surface roughness of the metasurfaces may be another issue that brings extra loss and decreases the transmission coefficients [30]. Besides, it is worth noting that the substrate effects, including losses and reflections, are discussed in detail in Additional file (see Additional file 1: Fig. S2). Even so, the similar variation trends between the measured and simulated results validate the performance of the dielectric metasurfaces for polarization control.

**Fig. 3:**
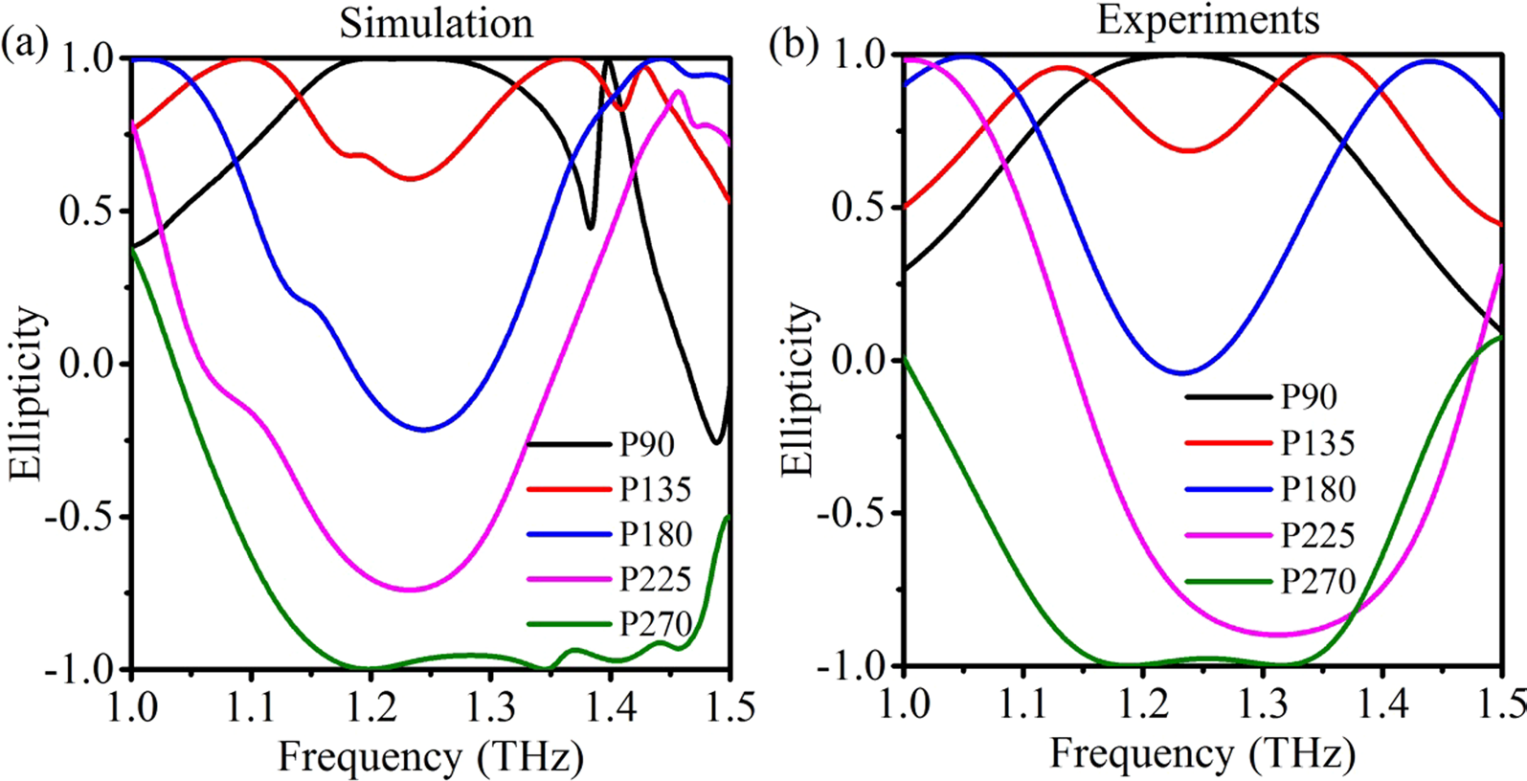
**a** Simulated and **b** experimental measured ellipticity of different dielectric metasurfaces

In order to fully investigate the performance of polarization conversion in the metasurfaces, the ellipticity of the transmitted wave was calculated, which is defined as:

3 $$\chi = S_{3} /S_{0} ,$$

where *S*_0_ and *S*_3_ are the Stokes parameters that can be directly calculated based on the transmission coefficients and phase delays [29]. As shown in Fig. 3, the simulation results present a full coverage of the ellipticity from 1 to − 1. Generally, the performance of polarization conversion close to 1.2–1.3 THz shows similar variation trends for both the simulation and experimental results. Some discrepancies occur at around 1.4 THz, which may originate from two aspects. First, in simulation, the substrate is treated as a lossless material with infinite thickness, while in experiments, the substrate possesses obvious losses with a thickness of 300 μm. These losses would damp the high-*Q* resonances (MD at 1.4 THz for example) and flatten the transmission spectra. Second, the geometric parameters of the resonators in experiments are varied compared to those defined in the simulation. A typical example is the width of the pillar that varies gradually at different heights, which attributes to the deep reactive ion etching process in fabrication. These geometric parameter variations would broaden the multipoles and increase their overlapping, and thus the individual high-*Q* resonances deteriorate due to the superposition and interference. In brief, the substrate effect and the geometry parameter variation in experiments collectively result in the discrepancies compared to those in the simulation at around 1.4 THz. Such discrepancies can be further minimized by choosing low loss substrates (e.g., quartz, polyimide, SU8) with small thickness and optimizing the fabrication process with respect to the simulated parameters. It is also noted that the operating frequencies were generally designed to be at off-resonance frequencies, hence weakly affected by the deterioration of high-*Q* resonance.

**Fig. 4:**
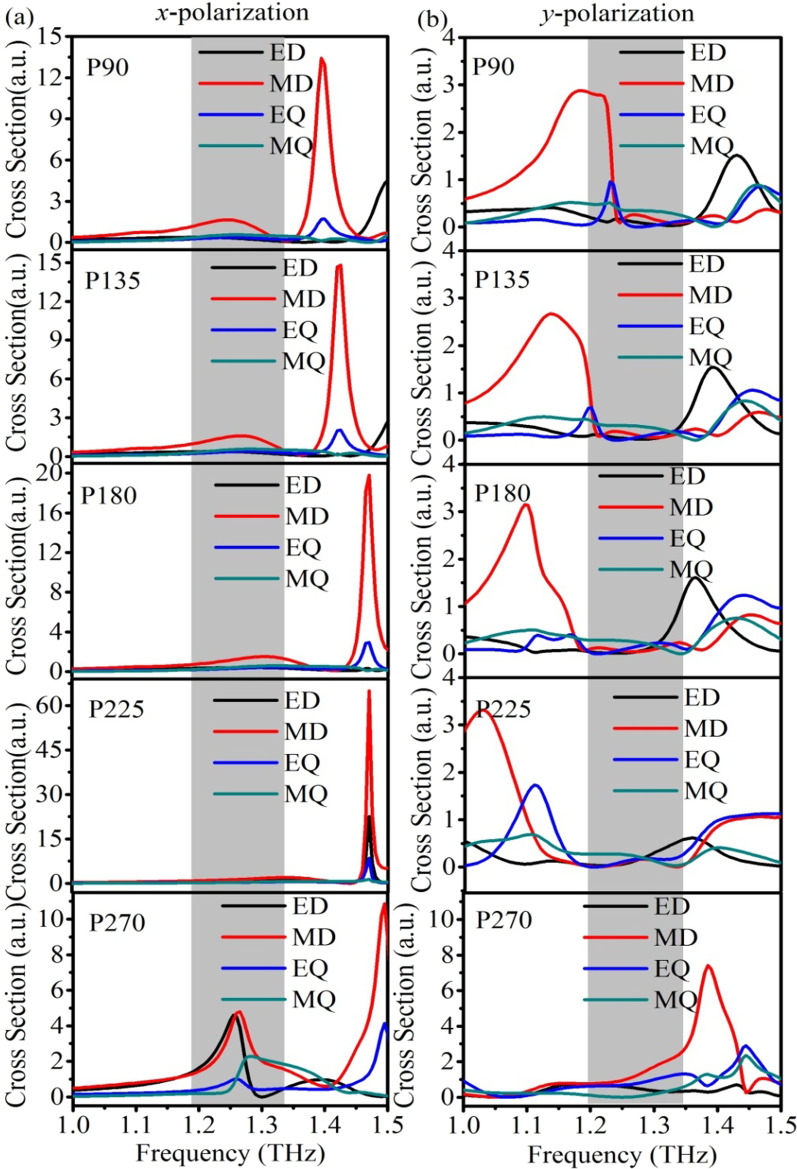
Multipole decomposition of the SCSs for the ED, MD, EQ and MQ resonances under **a** *x*- and **b** *y*-polarized incidences

To illustrate multipolar interference in the dielectric metasurfaces, the scattering cross sections (SCSs) of different multipoles are calculated by spherical multipole decomposition with respect to two orthogonal polarization directions [19, 24]. The details of multipole decomposition can be found in the Methods section. Figure 4 shows the calculated SCSs of different dielectric metasurfaces under *x*- and *y*-polarized incidences. First for P90, the magnetic dipole (MD) resonance contributes to the overall SCS at 1.4 THz under *x*-polarized incidence, whereas under *y*-polarized light it mainly occurs at 1.18 THz. At higher frequency region at around 1.42 THz, the electric dipole (ED), electric quadrupole (EQ) and magnetic quadrupole (MQ) components show obvious contributions to the SCSs under *y*-polarized light. When comparing the SCSs under *x*- and *y*- polarized incidences, in their overlapped region between 1.2 and 1.3 THz, the off-resonance conditions ensure high transmission coefficients, while the interference among different multipoles lifts different phase dispersion curves for two orthogonal polarization directions. With a proper balance among difference multipoles, a certain phase delay with high transmission coefficients and broadened bandwidths can be achieved, which in our case corresponds to the phase delay of 90°. For the cases of P135, P180 and P225, the contributions from ED, MD, EQ and MQ present similar variation trend as the case of P90 with subtle change of the resonance frequencies and mode overlapping, which clearly demonstrate the functionality of multipolar interference for the polarization control. On the contrary, for the case of P270, the phase delay of 270° requires giant phase dispersion with high transmission in a broad band, which can be hardly realized via the off-resonance condition. To resolve this issue, we design the in-resonance condition for the P270 case. Under *x*-polarized incidence, the resonance modes of ED, MD and MQ show obvious contributions to the SCSs between 1.2 and 1.3 THz. Under *y*- polarized incidence, the MD resonance dominates at 1.39 THz. Thus, the multipolar interference effects lead to high transmission in a broad band with 270° phase delay.

Compared with other existing designs, our proposed design enables a single layer platform for complete terahertz polarization control. More importantly, the phase delay of our design can be altered from 90° to 270°, covering different polarization states, including circular polarization, elliptical polarization and cross-linear polarization, which is challenging to be achieved in other existing designs (Table 1). Meanwhile, the bandwidth and efficiency of our design can outperform other existing single layer designs. It should be noted that although multilayer designs present better performance compared with our design, these multilayer meta-structures require complex design and fabrication processes, which restrict their applications in compact terahertz optical systems. In addition, our designs realize different polarization conversions, while most multilayer designs achieve limited phase delays with single polarization conversion function.

**Table 1:** Comparison of the proposed design with other terahertz polarization converters

References	Design	Functionality			Efficiency (%)	Bandwidth (%)	Phase delay
		linear to CP	Linear to cross	Linear to elliptical			
[31]	Mono-layer metallic MSs	√	×	×	45	12	90°
[29]	Mono-layer metallic MSs	√	×	×	30	5	90°
[32]	Mono-layer metallic MSs	×	√	×	4–9	11	/
[9]	Bi-layer metallic MSs	√	×	×	30	33	90°
[33]	Bi-layer metallic MSs	√	×	×	50–80	12	90°
[34]	Multi-layer metallic MSs	√	×	×	70	53	90°
[10]	Multi-layer metallic MSs	×	√	×	50–80	111	/
[35]	Si elliptical holes	√	×	×	50	3	90°
[18]	Double-unit Si pillars	√	√	×	13	5	90°, 180°
This work	Elliptical Si pillars	√	√	√	44–72	8–16	90°–270°

MS, metasurface; CP, circular polarization

## Conclusions

In summary, we have proposed and experimentally demonstrated complete terahertz polarization control with broadened bandwidth and high efficiency via all dielectric metasurfaces. Composed by elliptical silicon pillar arrays, the proposed metasurfaces realize equal and high transmission coefficients along the *x*- and *y*-axes, while their phase delay can be continuously tuned from 90° to 270° with a step size of 45°. The corresponding ellipticity changes from 1 to − 1, indicating a full coverage of different polarized light, including LCP light, elliptically polarized light, cross-polarized light and RCP light. On top of that, multipole decomposition results verify different contributions of multipoles for the polarization control. Such multipolar interference assisted dielectric metasurfaces promise an exotic strategy for implementing high performance terahertz functional polarization control devices.

## Methods

The fabrication of the dielectric metasurfaces involves standard photolithography and deep reactive ion etching. Firstly, an intrinsic silicon wafer with a thickness of 500 μm was bonded on a glass wafer (BF33, thickness 300 μm) through anodic bonding. The resistivity of the silicon wafer is beyond 5,000 Ω·cm to eliminate the absorption loss in silicon in the terahertz region. The silicon wafer was thinned to a thickness of 180 μm. Then, the wafer was cleaned by acetone and deionized wafer for 30 min. Next, photoresist AZ4620 was spin-coated on the wafer, followed by soft-baking at 100 °C for 10 min. After spin-coating, the elliptical arrays were patterned on the photoresist by photolithography (MA6) with an exposure time of 40 s, followed by photoresist development in the developer for 3 min. After that, a hard-baking process was performed at 110 °C for 5 min. The next step was silicon etching by deep reactive ion etching for 56 min. In the last, the remaining photoresist was cleaned by acetone, isopropanol and deionized water.

The dielectric metasurfaces were characterized in the THz-TDS. In this system, terahertz wave was generated from a home-made spintronic terahertz emitter, which was pumped by a 100 fs pulse laser at 800 nm with a repetition rate of 80 MHz. Then emitted terahertz wave was collimated and focused by four off-axis parabolic mirrors. The measured sample was positioned at the point where terahertz wave was focused with a beam diameter of around 3 mm. In order to fully characterize the polarization state of terahertz wave, two terahertz polarizers were placed before and after the sample to control the polarization. In the last, terahertz wave was detected by the electro-optic sampling technique, where a 1 mm thick ZnTe (110) electro-optic crystal was used for detection. The probe laser is from the same laser system for terahertz generation with a probe power of 20 mW. The characterization was performed at room temperature with a nitrogen gas environment to remove water absorption in terahertz region.

The multipole decomposition was carried out via the in-house developed Matlab code. Firstly, the electric field distribution $\overline{\user2{E}}_{{{\mathbf{inter}}}} \left( {\hat{\user2{r}}} \right)$ inside the elliptical silicon pillar was extracted from the numerical simulated results. Then, the current density $\overline{\user2{J}}\left( {\hat{\user2{r}}} \right)$ in the silicon pillar was derived as $\overline{\user2{J}}\left( {\hat{\user2{r}}} \right) = - i\omega \left[ {\overline{\varepsilon }\left( {\hat{\user2{r}}} \right) - \varepsilon_{0} } \right]\overline{\user2{E}}_{{{\mathbf{inter}}}} \left( {\hat{\user2{r}}} \right)$, where* ω* is the angular frequency, *ε*_0_ is the vacuum permittivity. Next, different current multipole moments can be decomposed as:

4 $$\overline{\user2{M}}^{\left( l \right)} = \frac{{\text{i}}}{{\left( {l - 1} \right)!\omega }}\smallint \overline{\user2{J}}\left( {\hat{\user2{r}}} \right)\underbrace {{{\varvec{rr}} \ldots {\varvec{r}}}}_{{l - 1{\text{terms}}}}{\text{d}}^{3} {\varvec{r}},$$

where *l* is the order of different moments and $\overline{\user2{M}}^{\left( l \right)}$ is a tensor of rank *l* [19, 24]. We calculated the first- and second-order current multipole moments, which correspond to dipole and quadrupole moments. Other high-order moments are not taken into account as they are generally very weak and make negligible contributions to the overall scattered fields. Based on the first- and second-order current multipole moments, multipole coefficients $a_{E} \left( l \right)$ and $a_{M} \left( l \right)$ can be obtained straightforwardly. Thus, the scattering cross sections of multipolar modes can be calculated using the following equations,

5 $$C_{s} = \frac{\pi }{{k^{2} }}\mathop \sum \limits_{l = 1}^{\infty } \left( {2l + 1} \right)\left[ {\left| {a_{E} \left( l \right)} \right|^{2} + \left| {a_{M} \left( l \right)} \right|^{2} } \right],$$

where *k* is the wave number.

## Supplementary Information

**Additional file 1**. Supplementary information accompanies this paper can be found in Additional file 1.

## Data Availability

The datasets used and/or analyzed during the current study are available from the corresponding author on reasonable request.

## Abbreviations

SEM: Scanning electron microscope
LCP: Left-handed circularly polarized
RCP: Right-handed circularly polarized
SCS: Scattering cross section
MD: Magnetic dipole
ED: Electric dipole
EQ: Electric quadrupole
MQ: Magnetic quadrupole
THz-TDS: Terahertz time domain spectroscope
