# Supplementary material for: Complete Terahertz Polarization Control with Broadened Bandwidth via Dielectric Metasurfaces
Source: Nanoscale Res Lett. 2021 Oct 19;16:157. doi: 10.1186/s11671-021-03614-y (PMC8526640; doi:10.1186/s11671-021-03614-y)
Supplement: Supplementary file 1 — Additional file 1. Supplementary information accompanies this paper can be found in Additional file 1. [file 11671_2021_3614_MOESM1_ESM.docx]

Supplementary information

This document provides supplementary information to “Complete terahertz polarization control with broadened bandwidth via dielectric metasurfaces” regarding a comparison of phase delay, a parameter table and discussion.

1. Comparison of Phase Delay

A single resonator in metallic metasurfaces can only induce π phase shift, as shown in Fig. S1(a). Dielectric metasurfaces with dual dipole modes can produce 2π phase shift, as shown in Fig. S1(b). When multipolar dielectric metasurfaces are designed to lift the phase dispersion with up to 4π phase shift, the phase delay beyond π and close to 2π between two orthogonal directions within a broad band can be achieved, as shown in Fig. S1(c). It is observed that with π phase shift, the phase delay would be smaller than π within a narrow band [1]. Similarly, with 2π phase shift, the phase delay would be smaller than 2π within a narrow band [2]. In these two cases, it is challenging to realize phase delay changing from 90° to 270° with broadened bandwidth and high efficiency. However, if the phase shift reaches 4π with the multipolar interference effect, it would be amenable to obtain giant phase delay with high efficiency and broad bandwidth.


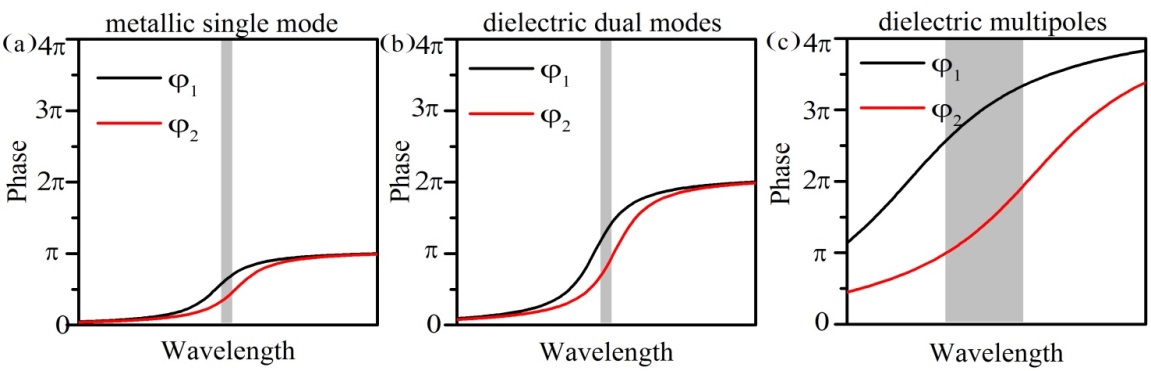


**Fig. S1** Phase shifts of **a** metallic resonators with a single mode and dielectric resonators with **b** dual dipole modes and **c** multipolar modes.

1. Parameter Table

Table S1 Parameters of five dielectric metasurfaces (μm).

| Designs | *a* | *b* | *h* | *P_x_* | *P_y_* |
| --- | --- | --- | --- | --- | --- |
| P270 | 38 | 140 | 180 | 100 | 200 |
| P225 | 41 | 80 | 180 | 100 | 170 |
| P180 | 43 | 74 | 180 | 100 | 140 |
| P135 | 47 | 66 | 180 | 100 | 140 |
| P90 | 50 | 60 | 180 | 100 | 140 |

1. Discussion

In the manuscript in Fig. 2, we presented the experimental measured transmission coefficients of the dielectric metasurfaces, which were normalized to a reference of the substrate. Such normalization eliminates the influence in the substrate and verifies the functionalities of polarization conversion in the silicon pillar arrays. In order to fully investigate the performance of the metasurfaces for practical applications and take the influence of the substrate into account, the transmission spectra of the metasurfaces normalized to air are shown in Fig. S2. It is observed that the main features of the transmission coefficients in Fig. S2 are similar to those shown in Fig. 2(b), except that the transmission coefficients are smaller. The discrepancy of the transmission coefficients is mainly attributed to losses in the substrate, which can be verified via the transmission coefficient of the bare substrate, as shown in Fig. S2. These losses brought by the substrate can be readily minimized by utilizing other dielectric materials with low refractive index, low loss and small thickness, such as quartz, polyimide, SU8, PDMS, etc. Anti-reflection coating at the bottom of the substrate can be another means to further enhance the transmission coefficients. Here, we performed the normalization with respect to the substrate in order to clearly validate the functionalities of the dielectric metasurfaces for polarization control without the influence of the substrate.


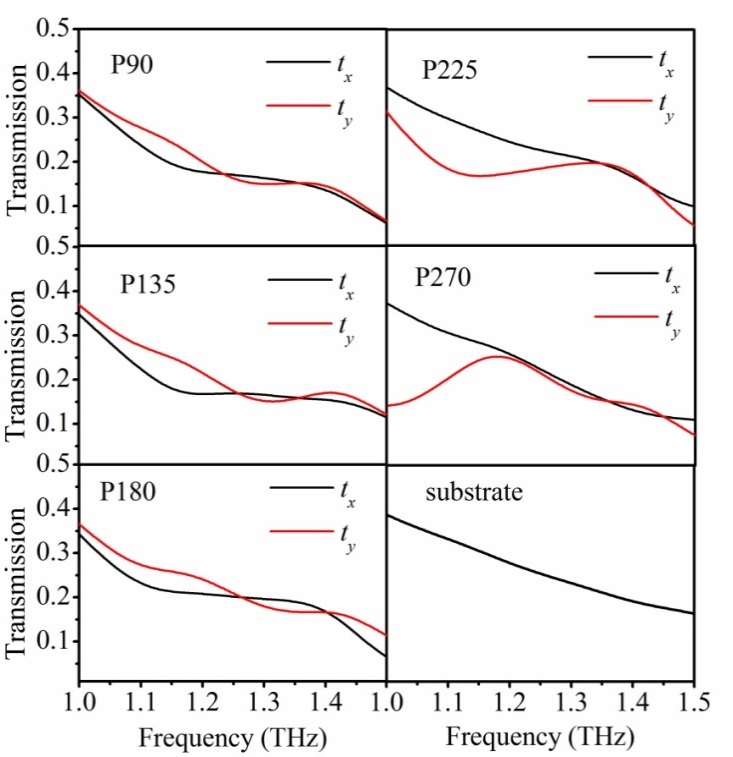


**Fig. S2** Experimentally measured transmission coefficients of dielectric metasurfaces for the cases of P90, P135, P180, P225, P270 and the bare substrate, respectively

References

1. Yu N, Aieta F, Genevet P, Kats MA, Gaburro Z, Capasso F (2012) A broadband, background-free quarter-wave plate based on plasmonic metasurfaces. Nano Lett 12(12): 6328-6333

2. Decker M, Staude I, Falkner M, Dominguez J, Neshev DN, Brener I, Pertsch T, Kivshar YS (2015) High-efficiency dielectric huygens’ surfaces. Adv Opt Mater 3(6): 813-820
